# Association of ambient temperature on acute ischemic stroke in Yancheng, China

**DOI:** 10.1186/s12889-024-19423-8

**Published:** 2024-07-15

**Authors:** Kai Qian, Qian Sun, Yanlong Li, Jin Chen

**Affiliations:** 1 Department of Neurology, Dongtai People’s Hospital, Yancheng, 224200 Jiangsu China; 2 Department of Respiratory Medicine, The First People’s Hospital of Yancheng, Affiliated Hospital of Nanjing University Medical School, Yancheng, 224006 Jiangsu Province China; 3 Department of Neurology, The First People’s Hospital of Yancheng, Affiliated Hospital of Nanjing University Medical School, No. 166 Yulong West Road, Yancheng, 224006 Jiangsu Province China

**Keywords:** Ambient temperature, Acute ischemic stroke, Hospitalizations, Time-series study

## Abstract

**Background:**

Acute ischemic stroke (AIS) is a major global public health issue. There is limited research on the relationship between ambient temperature and AIS hospital admissions, and the results are controversial. Our objective is to assess the short-term impact of ambient temperature on the risk of AIS hospital admissions in Yancheng, China.

**Methods:**

We collected data on daily AIS hospital admissions, meteorological factors, and air quality in Yancheng from 2014 to 2019. We used Poisson regression to fit generalized linear models and distributed lag non-linear models to explore the association between ambient temperature and AIS hospital admissions. The effects of these associations were evaluated by stratified analysis by sex and age.

**Results:**

From 2014 to 2019, we identified a total of 13,391 AIS hospital admissions. We observed that the influence of extreme cold and heat on admissions for AIS manifests immediately on the day of exposure and continues for a duration of 3–5 days. Compared to the optimal temperature (24.4 °C), the cumulative relative risk under extreme cold temperature (-1.3 °C) conditions with a lag of 0–5 days was 1.88 (95%CI: 1.28, 2.78), and under extreme heat temperature (30.5 °C) conditions with a lag of 0–5 days was 1.48 (95%CI: 1.26, 1.73).

**Conclusions:**

There is a non-linear association between ambient temperature and AIS hospital admission risk in Yancheng, China. Women and older patients are more vulnerable to non-optimal temperatures. Our findings may reveal the potential impact of climate change on the risk of AIS hospital admissions.

## Introduction

Acute Stroke, characterized by high morbidity, recurrence, disability, mortality and economic burden, is a serious public health issue [1]. According to statistics from the Global Burden of Diseases, Injuries, and Risk Factors Study (GBD), there were 12.2 million stroke cases, 101 million stroke endemic cases, 143 million DALYs duo to stroke, and 6.55 million stroke deaths in 2019 [2]. Acute ischemic stroke (AIS) remains the second leading cause of death and third leading cause of disability in the world and the first leading cause of death in China, which accounts for one-fifth of the world’s population. Ischemic stroke accounts for 69.6%~70.8% of all stroke hospitalized patients in China [2, 3]. AIS is serious and has poor prognosis, which brings a serious medical burden to individuals, families and society [4–6]. Risk factor management has been the cornerstone for prevention and therapy of AIS.

Previous epidemiological studies have revealed that seasonality constitutes an important risk factor for the development of AIS [7–9]. The Global Burden of Disease study reported that acute heat and cold exposure can increase the risk of mortality for AIS of causes of death [2, 10]. In Central Europe, the incidence of AIS increases with decreasing temperatures, and this trend is particularly pronounced among the elderly population [11]. Pedro and Leonardo et al. discovered that seasonal variation significantly impacts the rate of AIS hospitalizations in Brazil [7]. Furthermore, several studies have highlighted the extreme temperatures are a key factor leading to a rise in hospitalization and mortality rates associated with AIS in East Asian regions, specifically China, Japan, and South Korea [12–15]. Although previous studies have demonstrated a correlation between the incidence of AIS and temperature, these investigations primarily focus on Central Europe and East Asia, with varying results. These discrepancies may arise from differences in population characteristics, lifestyle habits, and climatic conditions across regions. As the largest developing country in the world, China faces significant public health challenges posed by climate change, including an increasing incidence and medical burden of various diseases, such as AIS [5]. However, research on the correlation between temperature and AIS in the eastern coastal cities of China remains limited.

Yancheng, as a typical coastal city, experiences distinct climatic influences, with cold air masses from the Eurasian continent in winter and the Pacific subtropical high in summer. Extreme temperatures are relatively common in Yancheng, with summer temperatures often exceeding 30 °C and winter temperatures dropping below 0 °C. These unique climatic characteristics and population structures may differentially impact the incidence of AIS. Therefore, understanding the relationship between temperature and AIS hospitalizations in Yancheng not only enhances the epidemiological study of AIS but also provides scientific evidence for developing more effective stroke prevention and intervention measures locally. In this study, we explored the association between ambient temperature and AIS during 2014–2019 in Yancheng, Jiangsu Province, a coastal city in eastern China.

## Materials and methods

### Study site

Located on the central and eastern coastline of Jiangsu Province in China, Yancheng spans an area of 16,931 km² and population of approximately 6.7 million. Geographically positioned between 32.85° and 34.2° north latitude, and 119.57° and 120.45° east longitude, it shares its eastern border with the Yellow Sea. Yancheng, located in the northern part of the northern subtropical zone, experiences a subtropical monsoon climate.

### Health data collection

We obtained daily AIS hospitalization records (January 1, 2014 to December 31, 2019) from the First People’s Hospital of Yancheng. Hospitalization for AIS was identified using the 10th revision of International Classification of Diseases (ICD-10) codes (I63.0-I63.9). The computerized medical record pertinent information about the patient, including admission and discharge dates, age, sex, and home addresses. This research project was approved by the Ethics Committee of the First People’s Hospital of Yancheng City.

### Environmental data collection

We acquired daily meteorological data on average temperature and relative humidity from the China Meteorological Data Sharing Service System (https://weather.cma.cn/) for the duration of our study. The meteorological data were measured at a fixed monitoring station approximately 2.4 km from the hospital. To verify the stability of the observed association after adjusting for air pollution factors, we extracted daily fine particulate matter (PM_2.5_), nitrogen dioxide (NO_2_) and sulfur dioxide (SO_2_), from the China’s National Urban Air Quality Real-time Publishing Platform (https://air.cnemc.cn:18007/). We extracted exposure data from five fixed environmental air quality monitoring stations in the urban area of Yancheng and calculated the average value as the daily mean concentration for each air pollutant. Meteorological data had approximately 0.31% missing values, and pollutant data had about 0.45% missing values. We handled these missing values by excluding them from the analysis using the ‘na.exclude’ function in our statistical analyses. This method ensures the analysis is conducted only on available data while preserving the original dataset structure. Patients residing more than 10 km from the monitoring station were excluded.

### Statistical analyses

We conducted a time-series design to explore the potential associations of ambient temperature and AIS hospital admissions. Since the daily hospital admissions approximately follow a Poisson distribution, we employed the generalized additive model (GAM) to investigate the association between daily mean temperature and AIS hospital admissions. GAM minimizes the occurrence of bias by controlling for seasonal, long-term trends and other confounding factors. Additionally, GAM facilitates the precise characterization of error patterns and is particularly effective with datasets exhibiting non-normal distributions, thereby ensuring lower and more dependable p-values. In this study, we also used a distributed lag non-linear model (DLNM) into the GAM to explore the association of ambient daily mean temperature and AIS hospital admissions [16]. The DLNM model has been widely used to examine the nonlinear and delayed effect between human health and environmental factors.

In line with prior studies [17–19], we adjusted several covariates in the model and selected degrees of freedom (df) in model based on the minimum Akaike information criterion statistic: (1) a natural spline of calendar time with 7 df per year to control for potential confounding by seasonal patterns and long-term time trends; (2) a natural spline with 3 df for daily mean relative humidity; (3) dummy variable and binary variable to control day of the week (DOW) effects and the public holiday effect, respectively; (4) natural splines (6df) of 3-day moving average PM_2.5_, SO_2_ and NO_2_ to control the confounding effects of air pollution. In addition, we incorporated a B-spline (2 degrees of 5 dfs) in the DLNM to address the delayed and non-linear impacts of daily mean temperature on AIS hospital admissions. A natural cubic spline with two internal knots, evenly spaced in log-lagged values, was employed for flexible representation of short-delay effects. A maximum lag of 21 days was used in the temperature’s cross-basis matrix to fully capture the entire lag structure and potential harvesting effect. The model is given below:

𝑌𝑡~𝑃𝑜𝑖𝑠𝑠𝑜𝑛(𝜇_𝑡 )

Log ($$\:{\mu\:}_{t}$$) = $$\:\alpha\:$$ + $$\:\beta\:{T}_{t,l}$$ + $$\:ns$$ (RH, $$\:df$$ = 3) + $$\:ns$$ (PM_2.5_, $$\:df$$ = 6) + $$\:ns$$ ($$\:{SO}_{2}$$_,_$$\:df$$ = 6) + $$\:ns$$ ($$\:{NO}_{2}$$, $$\:df=6)+$$$$\:ns(Time,\:df=7$$$$\:*year)+DOW+Holiday$$

where $\:{Y}_{t}$ is the daily number of AIS hospital admissions on day t; $\:{\mu\:}_{t}$ represents the expected daily AIS admissions for the same day; $\:\alpha\:$ is the intercept of the model; $\:{T}_{t,l}$ indicates the crossbasis matrix of temperature obtained from the DLNM; l indicates the lag days and $\:\beta\:$ indicates vector of coefficients for $\:{T}_{t,l}$; ns indicates the natural cubic spline function; $\:df$ indicates the degrees of freedom and $\:DOW$ is the indicator variable for day of week.

We firstly identified the minimum morbidity temperature, which is the temperature with the least impact on morbidity risk, as determined by the nadir of the aggregate cumulative exposure-response curve that correlates temperature with AIS. Subsequently, we explored the impacts of extreme cold (1th percentile, -1.3 °C) and heat (99th percentile, 30.5 °C) using the minimum morbidity temperature (24.4 °C) as a reference. Our model controlled for seasonal, long-term trends, as well as other confounding factors such as air pollution levels, day of the week, and holidays, to evaluate the associations between ambient temperature and AIS. To investigate the potential influence of demographic characteristics on AIS hospital admissions, we conducted subgroup analyses, stratifying the data by sex (male and female) and age (< 70 and ≥ 70 years). A Z-test was employed to evaluate the statistical significance of the differences observed between the estimates of these strata. Finally, we performed several sensitivity analyses to test the robustness of our findings: (1) evaluating the impact of alternative dfs (4–8 per year) for the time trend in the model, (2) adjusting for the effects of PM_2.5_, NO_2_, and SO_2_ and (3) using three internal knots to adjust the lag structure of temperature.

All statistical analyses were performed in R software (version 4.3.2) using the ‘dlnm’, ‘mgcv’, and ‘splines’ packages. We conducted statistical tests using a two-sided approach, p-value < 0.05 was considered statistically significant for model fitting and effect modification.

## Results

Table 1 summarizes the descriptive results. During the study period, a total of 13,391 patients were hospitalized due to AIS, with an average of 6 patients per day. Male and female patients accounted for 57.9% and 42.1%, respectively. Patients aged over 70 years comprised 55.7%, while those under 70 years constituted 44.3%. As shown in Table 1, the daily average temperature and relative humidity (± SD) were 15.0 °C (± 9.0 °C) and 79.4% (± 12.1%), respectively, during the study period. Regarding air pollution, the average daily concentrations (± SD) of PM2.5, SO2, and NO2 were 45.7 (± 32.3) µg/m³, 14.1 (± 9.5) µg/m³, and 24.6 (± 12.6) µg/m³, respectively.

**Table 1:** Descriptive statistic of AIS hospital admissions and environmental variables in Yancheng, China, 2014–2019

Variables		Counts	Mean	SD	Min	P25	P50	P75	Max
AIS patients									
Total		13,391	6	3	0	4	6	8	23
Sex	Man	7731	4	2	0	2	3	5	14
	Female	5660	3	2	0	1	2	4	15
Age	< 70 years	5886	3	2	0	1	2	4	14
	≥ 70 years	7505	3	2	0	2	3	5	16
Weather conditions									
Mean temperature(°C)		2184	15.0	9.0	-4.7	6.9	15.7	22.9	32.9
Relative humidity (%)		2184	79.4	12.1	34.2	71.3	81.8	88.3	100.0
Air pollutants									
PM_2.5_ (µg/m^3^)		2181	45.7	32.3	5.0	23.1	35.9	58.8	230.0
SO_2_ (µg/m^3^)		2181	14.1	9.5	2.0	8.2	11.9	17.0	80.1
NO_2_ (µg/m^3^)		2181	24.6	12.6	3.0	15.7	21.5	30.3	87.9

Abbreviations : AIS: Acute ischemic stroke; PM_2.5_: Particles with aerodynamic diameter < 2.5 μm; SO_2_: Sulfur dioxide; NO_2_: Nitrogen dioxide; SD: Standard deviation

**Fig. 1:**
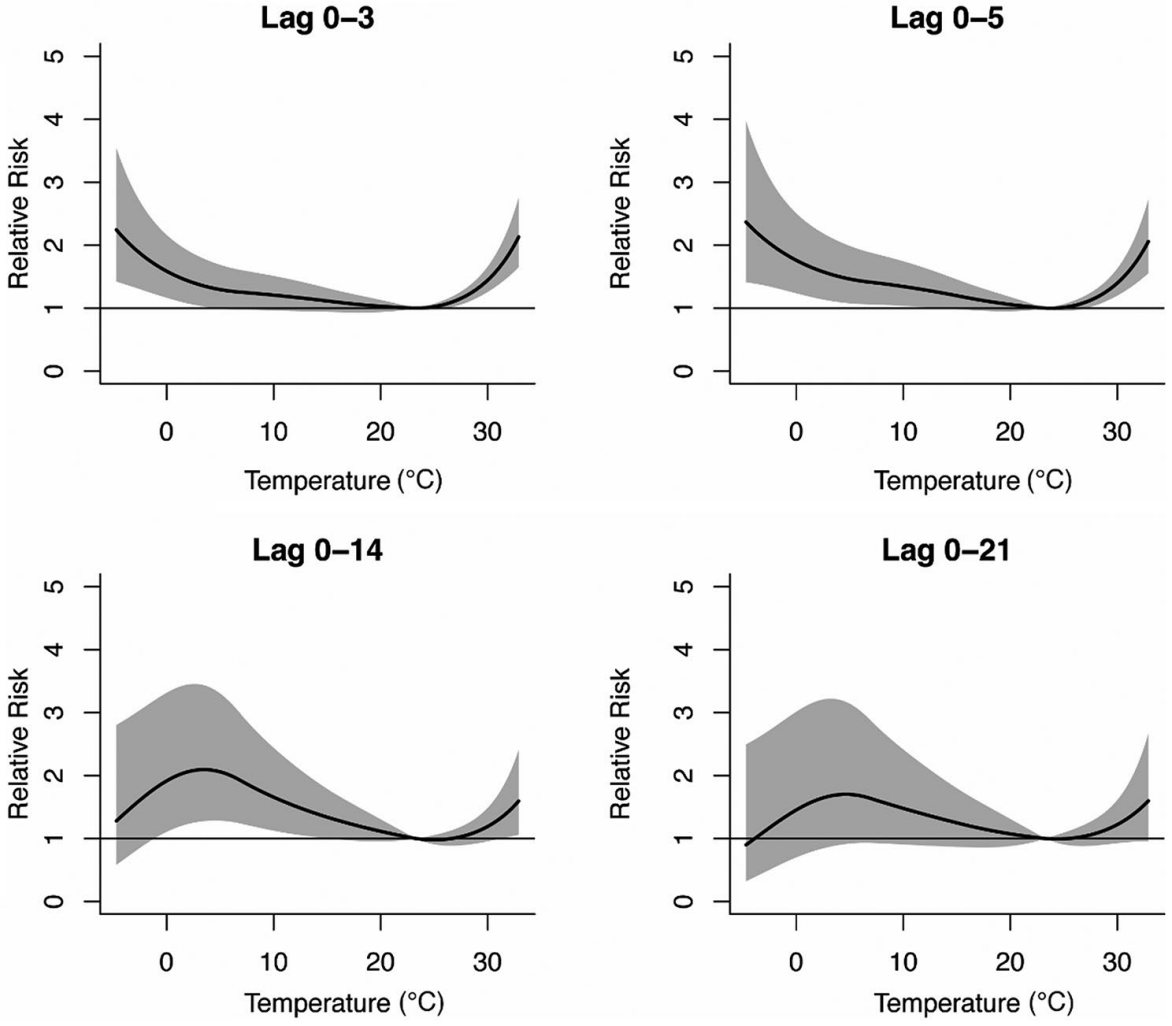
The cumulative exposure–response relationship curves between AIS and daily mean temperature in Yancheng over lag 0–3, 0–5, 0–14 and 0–21 days. The referent temperature was 24.4 °C. The black solid lines are estimate of RRs and the gray areas are the 95% confidence intervals

Figure 1 illustrates the exposure-response relationship curves between daily average temperature and daily AIS hospital admissions for different lag days (Lag0-3, Lag0-5, Lag0-14, Lag0-21). Generally, these curves are similar in shape and reference temperature, consistently exhibiting an inverse “J-shape”. On lag 0–3 and lag 0–5, the curves rise slowly as the average temperature decreases from the reference temperature (24.4 °C) to 0 °C, but then sharply ascends below 0 °C. Notably, as the daily average temperature increases from the reference temperature (24.4 °C), the curves show a sharp linear rise.

**Fig. 2:**
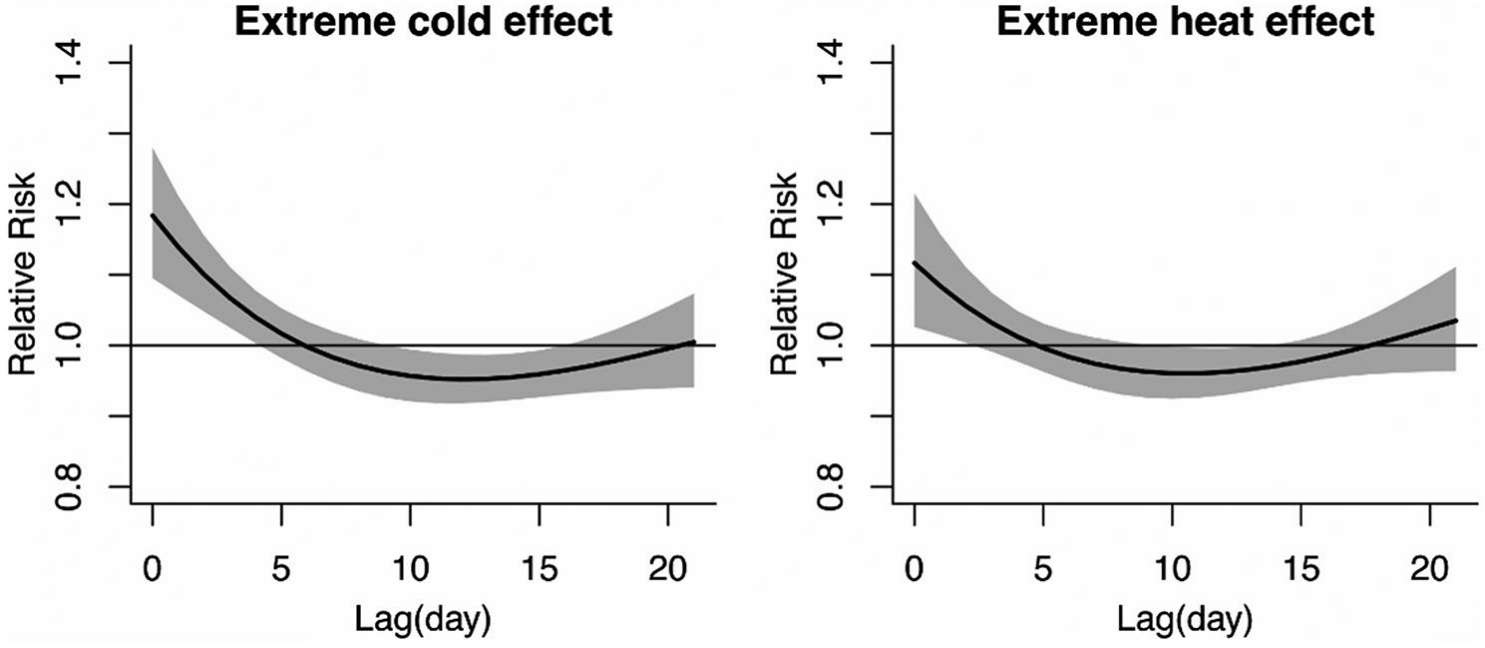
The lag structures in the effects of extreme temperatures (Left: -1.3 °C, 1st percentile of temperatures; Right: 30.5 °C, 99th percentile of temperatures) on hospital admissions for AIS. The referent temperature was 24.4 °C. The black solid lines are estimate of RRs and the gray areas are the 95% confidence intervals

Figure 2 illustrates the lag pattern (lag 0–21 days) of the impact of extreme cold (-1.3 °C) and extreme heat (30.5 °C) on hospital admissions for AIS. The risk of hospitalization due to extreme cold temperatures and extreme heat is strongest on the same day (lag0), attenuated drastically to lag day 5, and then shows a notable displacement (relative risk below 1.0). Furthermore, we observed that from lag day 10 to 15, the impact of extreme cold significantly decreases (also known as the ‘harvesting effect’).

Table 2 shows the pooled RRs for AIS hospital admissions under extreme cold and heat temperature levels during different lag periods compared to the reference temperature (24.4 °C). Specifically, under extreme cold temperatures, the RRs for total AIS hospitalizations at lag0-3 and lag0-5 are 1.70 (95%CI: 1.21, 2.40) and 1.88 (95%CI: 1.28, 2.78), respectively. The RRs under extreme heat temperatures are 1.51 (95%CI: 1.31, 1.75) and 1.48 (95%CI: 1.26, 1.73). Furthermore, we found that the impact of extreme cold on AIS was more pronounced in female patients, with RRs of 1.93 (95%CI: 1.17, 3.17) and 1.94 (95%CI: 1.10, 3.42) at lag0-3 and lag0-5, respectively, although the difference was not significant (*P*_Lag0−3_ = 0.51 and *P*_Lag0−5_ = 0.81). During exposure to extreme cold temperatures, the estimated values were only significant for elderly patients, with cumulative RRs of 2.51 (95%CI: 1.54, 4.09) and 2.56 (95%CI: 1.47, 4.46) at lag0-3 and lag0-5, respectively, but again the difference was not significant. Similarly, we observed the same phenomenon under extreme high temperatures, with a more significant impact on females and elderly patients. Generally, females and elderly patients are more susceptible to the effects of extreme low and high temperatures.

**Table 2:** The cumulative relative risks (RRs) for daily AIS hospital admissions associated with extreme cold temperature (-1.3 °C, 1st percentile of temperatures) and extreme heat temperature (30.5 ° C, 99th percentile of temperatures), relative to the reference temperature of 24.4 °C

Lag days	Variables	Extreme cold	Extreme heat
Lag 0–3	Total	**1.70 (1.21**,** 2.40)**	**1.51 (1.31**,** 1.75)**
	Male	1.44 (0.93, 2.20)	**1.36 (1.14**,** 1.61)**
	Female	**1.93 (1.17**,** 3.17)**	**1.83 (1.48**,** 2.26)**
	*P* for effect modification	0.51	0.06
	< 70 years	1.04 (0.64, 1.71)	**1.31 (1.06**,** 1.62)**
	≥ 70 years	**2.51 (1.54**,** 4.09)**	**1.66 (1.36**,** 2.03)**
	*P* for effect modification	0.09	0.15
Lag 0–5	Total	**1.88 (1.28**,** 2.78)**	**1.48 (1.26**,** 1.73)**
	Male	**1.71 (1.05**,** 2.79)**	**1.31 (1.09**,** 1.58)**
	Female	**1.94 (1.10**,** 3.42)**	**1.82 (1.44**,** 2.29)**
	*P* for effect modification	0.81	0.07
	< 70 years	1.28 (0.73, 2.25)	**1.29 (1.02**,** 1.62)**
	≥ 70 years	**2.56 (1.47**,** 4.46)**	**1.62 (1.30**,** 2.01)**
	*P* for effect modification	0.24	0.21
Lag 0–14	Total	1.80 (0.99, 3.30)	**1.27 (1.01**,** 1.59)**
	Male	1.26 (0.59, 2.70)	1.12 (0.85, 1.48)
	Female	2.34 (0.98, 5.60)	**1.57 (1.13**,** 2.22)**
	*P* for effect modification	0.55	0.21
	< 70 years	2.07 (0.86, 4.96)	1.19 (0.86, 1.64)
	≥ 70 years	1.71 (0.96, 4.03)	1.32 (0.96, 1.84)
	*P* for effect modification	0.85	0.69
Lag 0–21	Total	1.32 (0.60, 2.95)	1.28 (0.96, 1.70)
	Male	0.89 (0.32, 2.46)	1.20 (0.85, 1.69)
	Female	1.80 (0.56, 5.73)	1.48 (0.97, 2.27)
	*P* for effect modification	0.68	0.55
	< 70 years	0.53 (0.17, 1.68)	1.23 (0.82, 1.84)
	≥ 70 years	2.84 (0.91, 8.89)	1.29 (0.86, 1.93)
	*P* for effect modification	0.46	0.89

Abbreviations: Statistically significant results (*p* < 0.05) are highlighted in bold

## Discussion

In this time series study, we employed DLNM to investigate the association between AIS and ambient temperature in the Yancheng region of China from 2014 to 2019. We observed that the risk of hospitalization for AIS was associated with both extremely low and high temperatures. Additionally, we found that women and elderly patients aged 70 and above were more susceptible to temperature-related risk factors. Our findings provide rare epidemiological evidence for the impact of extreme temperatures in subtropical monsoon regions on AIS hospitalizations and carry broader public health implications for the prevention of temperature-induced AIS.

The nonlinear relationship between temperature and AIS identified in this study has been confirmed by many previous global studies. A study in Kaunas, Lithuania, found that for each additional cold day in the week preceding a stroke, the risk of stroke increased by 3%, and in summer, this risk increased to 8% [9]. A similar study in Beijing, China, reported that extreme cold temperature (relative to 25th percentile of temperature (1.2 °C)) were associated with an increased rate of AIS hospital admissions[21]. Another study in Hefei, China, found that the cumulative effect of extreme cold on the risk of AIS reached its maximum at a lag of 0–21 days, with an RR of 2.378 (95% CI: 1.304, 4.337) [13]. On the other hand, some studies indicate a positive correlation between high temperatures and AIS hospital admissions. A study in Nanchang, China, found that exposure to extreme high temperatures at lag0-3 was associated with the risk of admission for ischemic stroke, with an RR of 1.18 (95% CI: 1.07, 1.36) [21]. A similar study in Shenzhen, China, reported that higher temperatures (relative to the 10th percentile of median temperatures) were associated with an increased rate of AIS hospital admissions [22]. A case-crossover study conducted in Brisbane, Australia, found that high temperatures had a significant impact on stroke admissions among individuals with hyperlipidemia (RR: 1.85; 95% CI: 1.07, 3.19) [23]. Similar to our findings, a time-series study conducted in 11 cities in Jiangsu, China, observed a generally “U”-shaped association curve between temperature and stroke mortality, with relative risks for extreme low and high temperatures of 1.31 (95% CI: 1.09, 1.57) and 1.62 (95% CI: 1.39, 1.88), respectively [24]. Variations in climate conditions, economic circumstances, lifestyle habits, and study designs may contribute to the inconsistency in findings across various studies. The climate of the Yancheng region is significantly influenced by maritime factors, leading to temperatures that are higher in summer and lower in winter compared to other regions at the same latitude. This is due to the influence of the Pacific subtropical high, which exacerbates summer heat, and the impact of Eurasian continental cold air masses, which intensify winter cold. This distinct climatic dichotomy likely contributes to the significant effects of both extreme heat and cold seasons on the incidence of hospital admissions for AIS.

The underlying biological mechanisms contributing to the burden of hospital admissions for AIS due to extreme heat and cold remain unclear. During cold conditions, alterations in coagulation function, changes in lipid and glucose levels, and modifications in inflammatory responses may increase the risk of AIS incidence and mortality [18, 25–27]. When exposed to high temperatures, the body may attempt to mitigate adverse effects through mechanisms such as sweating, vasodilation, and increased heart rate to facilitate heat dissipation. This can lead to dehydration, increased blood viscosity, elevated cholesterol levels, and a higher likelihood of thrombosis [23, 28]. Furthermore, exposure to extreme temperatures may result in heightened sympathetic nervous activity, leading to an increase in platelet count and blood viscosity, both of which could elevate an individual’s susceptibility to AIS [29].

Identifying the differential lagged effects of extreme temperatures is crucial for policymakers to formulate preemptive strategies against the adverse repercussions of climate change. In our study, we observed that the influence of extreme cold and heat on admissions for AIS manifests immediately on the day of admission and continues for a duration of 3–5 days. The lag period for evaluating the health effects of extreme temperature exposure has not yet been definitively established. Most studies demonstrate that the lagged effects of extreme heat manifest within a week [18, 22, 30], a conclusion consistent with our findings. Regarding the lag time associated with cold effects, there is notable inconsistency, with some studies indicating prolonged health implications lasting over two weeks or more [13, 18], while others find these effects are limited to a week or even shorter [19, 31]. The differences in lag times reported across studies may stem from the different methodologies and parameters researchers choose during the process of fitting their models. Additionally, the potential environmental and physiological mechanisms behind the lagged responses to extreme heat and cold remain to be elucidated. Regarding environmental factors, evidence indicates that factors such as geographical location, climatic conditions, ethnicity, socioeconomic status, levels of education, availability of green spaces, and air conditioning usage may alter the lagged health effects of extreme temperature exposure [18, 32, 33]. In summary, future research is hoped to focus on identifying optimal lag times and elucidating mechanisms behind the varied lag effects, enhancing our understanding of temperature-related health impacts.

Our sex-based subgroup analysis revealed that female patients with AIS are more vulnerable to the impacts of environmental temperatures compared to males, a finding consistent with several epidemiological studies [13, 22]. This sex disparity in temperature sensitivity could be attributed to physiological differences, including variations in sex hormones, thermoregulatory capabilities, and immune functions [34, 35]. Additionally, our age-based subgroup analysis indicated that the elderly (≥ 70 years) is disproportionately impacted by environmental temperatures compared to younger individuals, a finding that is also confirmed by previous studies [11, 36]. This discrepancy might be related to the higher prevalence of chronic comorbidities in older adults and a reduction in thermoregulatory efficiency and physiological adaptability as they age.

This study is subject to several limitations. First, the temperature data were derived from stationary monitoring sites, not personal exposure metrics, potentially introducing inaccuracies in estimating effects. Second, the study’s limitation to a single major hospital in one city results in a smaller sample size, potentially missing crucial correlations between temperature fluctuations and AIS, especially within subgroup analyses. Third, due to regional limitations, our study’s findings may not be applicable to areas with differing climatic zones or socio-economic environments. Additionally, this study did not assess the differential impact of individual characteristics such as the patient’s education level, socioeconomic status, availability of green spaces, air conditioning usage, current health status, and whether the hospitalization was for a first-time or recurrent AIS event. These factors could provide insights into whether certain individuals are less susceptible to the adverse effects of extreme temperatures.

Despite these limitations, our study has several strengths. First, we utilized a comprehensive dataset spanning six years, enhancing the robustness and reliability of our findings. Second, the application of advanced statistical models, such as the DLNM, enabled us to capture the complex, non-linear relationships between ambient temperature and AIS hospital admissions, accounting for both immediate and delayed effects. Third, our stratified analyses revealed insights into the vulnerability of specific subgroups, including women and the elderly, to temperature-related AIS risks. Finally, this study focuses on the impact of ambient temperature on AIS in Yancheng, a subtropical monsoon climate region, providing valuable localized data that can inform public health strategies in Yancheng and other regions with similar climatic conditions.

## Conclusions

In summary, this study demonstrates a nonlinear association between temperature exposure and AIS hospital admissions in Yancheng, China. Vulnerable groups, including women and the elderly, should be made more aware of the adverse effects of extreme temperatures. Our findings indicate that extreme temperatures should be considered a potential risk factor for AIS hospitalizations. These findings support the development and implementation of appropriate health risk education and effective intervention measures. Additionally, larger-scale multi-city studies are needed to further explore the impact of extreme temperatures on human health.

## Data Availability

The data that support the findings of this study are available from the corresponding author upon reasonable request.
